# Dietary Cadmium Intake and the Risk of Cancer: A Meta-Analysis

**DOI:** 10.1371/journal.pone.0075087

**Published:** 2013-09-17

**Authors:** Young Ae Cho, Jeongseon Kim, Hae Dong Woo, Moonsu Kang

**Affiliations:** 1 Molecular Epidemiology Branch, National Cancer Center, Goyang, Korea; 2 Department of Information Statistics, Gangneung-Wonju National University, Gangneung, Korea; Nanjing Medical University, China

## Abstract

**Background:**

Diet is a major source of cadmium intake among the non-smoking general population. Recent studies have determined that cadmium exposure may produce adverse health effects at lower exposure levels than previously predicted. We conducted a meta-analysis to combine and analyze the results of previous studies that have investigated the association of dietary cadmium intake and cancer risk.

**Methods:**

We searched PubMed, EMBASE, and MEDLINE database for case-control and cohort studies that assessed the association of dietary cadmium intake and cancer risk. We performed a meta-analysis using eight eligible studies to summarize the data and summary relative risks (RRs) and 95% confidence intervals (CIs) were calculated using a random effects model.

**Results:**

Overall, dietary cadmium intake showed no statistically significant association with cancer risk (RR = 1.10; 95% CI: 0.99–1.22, for highest vs. lowest dietary cadmium group). However, there was strong evidence of heterogeneity, and subgroup analyses were conducted using the study design, geographical location, and cancer type. In subgroup analyses, the positive associations between dietary cadmium intake and cancer risk were observed among studies with Western populations (RR = 1.15; 95% CI: 1.08–1.23) and studies investigating some hormone-related cancers (prostate, breast, and endometrial cancers).

**Conclusion:**

Our analysis found a positive association between dietary cadmium intake and cancer risk among studies conducted in Western countries, particularly with hormone-related cancers. Additional experimental and epidemiological studies are required to verify our findings.

## Introduction

Cadmium has been recognized as a carcinogen for many decades based on studies of occupationally exposed individuals [1]. Findings from earlier studies were based on substantial exposure via the respiratory system and indicated an adverse role of cadmium in the development of cancer [2]. However, the results of studies investigating the effect of low cadmium levels on human carcinogenesis are inconsistent. Because cadmium is almost ubiquitously present in the environment, there is growing concern about chronic exposure to low levels of cadmium. Recent studies have investigated the effect of cadmium exposure in the general population and suggested that cadmium may cause adverse health effects at lower exposure levels than previously expected [3].

Diet is the main source of environmental cadmium among non-occupationally exposed non-smokers [3]. Additionally, drinking water contributes only a very small percentage of a person’s total cadmium intake [4]. Based on estimates of cadmium intake, it has been reported that more than 80% of food-based cadmium comes from cereals and vegetables [4]. The average cadmium intake from food generally varies between 8 and 25 µg/day [3]. The gastrointestinal absorption of cadmium is much lower than the inhalation absorption of cadmium; it is estimated to be approximately 5% of an ingested amount of cadmium, depending on the nutritional status [5]. Once absorbed, cadmium binds to metallothionein and is stored mainly in the kidneys, liver and other organs. Its long biological half-life (10-30 years) in humans may lead to neoplastic transformation through multiple pathways [3,6,7]. However, the amount of exposure may differ among individuals based on their living environment, dietary habits, and cadmium absorption rate [3]. Therefore, dietary cadmium intake could be a risk factor for cancer among certain population subgroups [8].

Experimental studies using *in vitro* cell culture and *in vivo* animal studies demonstrated that cadmium exposure results in cell transformation and induces cancer in various organs [7]. Recently, several epidemiological studies have investigated the effect of dietary cadmium on cancer risk [9-16], but these findings are still inconsistent. Therefore, we aimed to combine and analyze the results of these existing studies.

## Methods

### Study Selection

We identified studies examining the association between dietary cadmium intake and cancer risk by searching the database of PubMed, EMBASE, and MEDLINE published up until May 2013. We used the following terms: (cancer OR carcinoma) AND (diet OR dietary) AND cadmium. There were no language restrictions. We also searched reference lists of the identified papers and of recent reviews to retrieve additional studies. In our meta-analysis, the following inclusion criteria were used for selecting the studies: 1) study design was either case-control or cohort studies; 2) the exposure to cadmium was measured through dietary intake; and 3) the primary outcome was cancer incidence. Cross-sectional studies, ecologic analyses, studies without informative effect estimates, studies using cadmium biomarkers, and duplicated studies were excluded from our meta-analysis.

**Figure 1:**
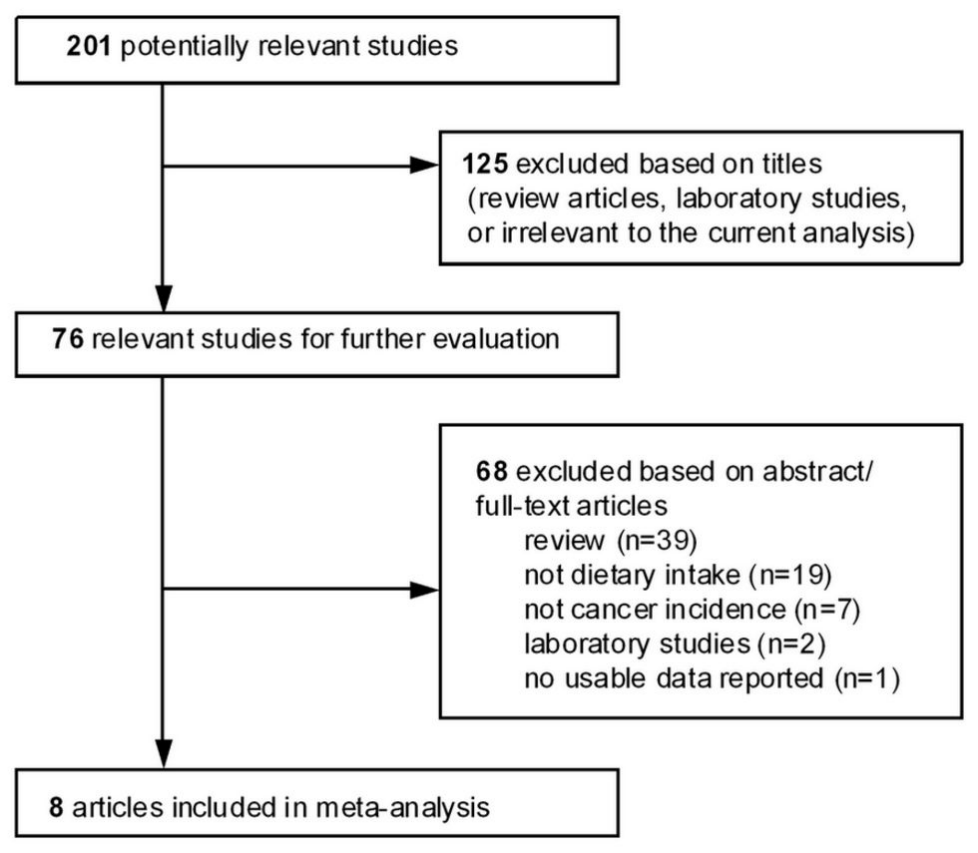
Study flow chart depicting literature search and selection.

We assessed the relevance of the studies using a hierarchical approach based on title, abstract, and full-text article. The study flow chart depicting the literature search and selection is presented in Figure **1**. Using the search terms mentioned above, a total of 201 articles were retrieved. We screened the titles of these articles and excluded 125 articles based on our inclusion and exclusion criteria. Then we examined the abstracts of the screened articles; full-texts were investigated if study eligibility was uncertain based on the abstracts. The articles were excluded for the following reasons; 1) reviews (n=39); 2) studies did not use dietary cadmium intake as exposure (n=19); 3) the primary outcome was not cancer incidence (n=7); 4) laboratory studies (n=2); and 5) an article did not report effect estimates (n=1). In addition, the related reference and review articles were searched to identify other relevant publications, but no additional manuscripts were included. Our final meta-analysis consisted of eight articles, consisting of two case-control studies and six cohort studies. Two of the authors independently identified and reviewed each relevant paper, and discrepancies in study eligibility were resolved by consensus.

### Data Extraction

Two authors independently extracted data in four categories from each eligible study as follows: 1) study description [cancer type; study type; total number of case and control subjects and/or cohort size, follow-up period, number of cases]; 2) exposure [method of dietary assessment, mean cadmium intake and range, scales used in the analysis, source of dietary cadmium if possible]; 3) outcome [adjusted odds ratio (OR), relative risk (RR), or hazard ratio (HR) for extreme comparison groups and 95% confidence intervals (CIs) for cancer incidence]; and 4) covariates used in multivariate analysis

### Data Analysis

All statistical analyses were performed using STATA software (version 11; Stata Corporation, College Station, Texas), and two-sided *P*-values less than 0.05 were considered statistically significant. We estimated the summary association between dietary cadmium intake and cancer incidence using a random effects model. The Q-statistic and I^2^ statistics were calculated to estimate heterogeneity. Potential sources of heterogeneity were determined via subgroup or sensitivity analyses. The data were stratified by study design (case-control/cohort), geographic location (Western/Asian), and cancer type (prostate cancer/breast cancer/endometrial cancer). We also performed sensitivity analyses to determine the effects of individual studies on the summary estimate by repeating the meta-analysis. For each subgroup analyses, we used a fixed or random effects model based on the results of the Q-statistics to calculate the summary RRs and 95% CIs [17]; we used a random effects model if *P* < 0.05 for heterogeneity. We also conducted a cumulative meta-analysis to evaluate whether the trend of summary RRs (95% CIs) changed over time as more data were collected. Studies were added one at a time according to the publication year. The results were summarized as each new study was added. Publication bias was examined using analyses described by Egger [18] and Begg [19] and a visual inspection of the resulting funnel plot.

## Results

Overall, the meta-analysis conducted in this manuscript contained eight studies, including two case-control studies (746 cases and 1069 controls) [15,16] and six cohort studies (309,103 participants and 12,859 cancer cases) [9-14]. The characteristics of included case-control studies and cohort studies are listed in Table **1** and Table **2**, respectively. Case-control studies were published in 1991 and 2013 [16], which were conducted in the United States and Japan. All cohort studies were published after 2008. Among these cohort studies, four were conducted in Sweden [10-13], two were conducted in the United States [9], and one was conducted in Japan [14]. Most studies investigated the role of cadmium in hormone-related cancers (endometrial, ovarian, breast, and prostate cancers), whereas one Japanese study examined various types of cancers [14]. To estimate dietary cadmium intake, all studies used the Food Frequency Questionnaire (FFQ). Major sources of cadmium are vegetables and cereal in Western populations, but rice was the primary cadmium source in the Japanese study. Most studies conducted subgroup analyses based on potential confounders.

**Table 1:** Characteristics of published case-control studies on the association between dietary cadmium intake and cancer risk in this meta-analysis.

**Study (Country**)	**Study description**	**Dietary cadmium intake (µg/day**)	**Outcome**	**Variables used in multivariate model**
West et al. (1991) USA	Prostate cancer; 358 cases/679 controls for all subjects; population-based control matched by age and county of residence	FFQ; Q1 (<36), Q2 (36–48), Q3 (49-61), Q4 (>61)	[Q4 vs. Q1] OR = 1.35 (0.94–1.96) for all subjects; OR = 1.12 (0.66–1.89) for men aged 45-67yr; OR = 1.82 (1.07–3.10) for men aged 68-74yr	None
Itoh et al. (2013) Japan	Breast cancer, 390 cases/390 controls; healthy control matched by age and residential area.	FFQ; 136-item semi-quantitative FFQ; Tertile median cadmium intake, T1 (21.4), T2 (26.2), T3 (31.5); the mean estimated energy-adjusted cadmium intake = 26.4 µg/day	[T3 vs. T1] OR=1.23 (0.76, 2.00) for all subjects; OR=1.94 (1.04-3.63) for postmenopausal women with ER+ tumor; OR=0.31 (0.13, 0.72) for BMI <21kg/m^2^; OR=2.30 (1.17, 4.52) for BMI 21−<25kg/m^2^; OR=2.42 (0.86, 6.82) for BMI ≥25kg/m^2^; Subgroup analyses by menopausal and smoking status were not significant.	Matched for age and residential area; physical activity, smoking, family history of breast cancer, number of births, isoflavone intake, vegetable intake, total energy intake; menopausal status if applicable.

ER, estrogen receptor; FFQ, food-frequency questionnaire; F/U, follow-up; Q, quartile; T, tertile; OR, odds ratio; RR, rate ratio; HR, hazard ratio

**Table 2:** Characteristics of published cohort studies on the association between dietary cadmium intake and cancer risk in this meta-analysis.

**Study (Country**)	**Study description**	**Dietary cadmium intake (µg/day**)	**Outcome**	**Variables used in multivariate model**
Akesson et al. (2008) Sweden	Endometrial cancer; the Swedish Mammography Cohort, 30,210 postmenopausal women; 16yr F/U, 378 cases	FFQ; 67-item, 96-item FFQ; The average estimated dietary cadmium intake = 15 µg/day (80% from cereals and vegetables); T1 (<13.7), T2 (13.7–16), T3 (≥16)	[T3 vs. T1] RR = 1.39 (1.04–1.86) for all women; RR = 1.86 (1.13–3.08) for non-smoker & BMI <27 kg/m^2^; RR = 2.42 (1.12-5.21) for non-smoker, BMI <27 kg/m^2^ and nonusers of postmenopausal hormones	Age, education, parity, age at menarche, age at menopause, leisure time physical inactivity, BMI, postmenopausal hormones use and smoking status.
Julin et al. (2011) Sweden	Ovarian cancer; the Swedish Mammography Cohort; 60,889; women; 18.9yr F/U; 409 cases	FFQ; 67-item, 96-item FFQ; T1 (<14), T2 (14–16), T2 (>16)	[T3 vs. T1] RR = 0.89 (0.70–1.14) for all subjects; None of the subgroup analyses were significant (BMI, smoking, postmenopausal hormone use, oral contraceptive use)	Age, BMI, education, age at menarche, use of oral contraceptives, age at menopause, postmenopausal hormone use, parity and age at first birth.
Admas et al. (2012) USA	Breast cancer; 30, 543 postmenopausal women; 7.5yr F/U 1,026 cases; VITamins And Lifestyle (VITAL) cohort	FFQ; 120-item FFQ; dietary cadmium intake=10.9 µg/day (vegetable 44%, grain 22%); Q1 (<7.48), Q2 (7.48–10.05), Q3 (10.06–13.30), Q4 (>13.30)	[Q4 vs. Q1] HR = 1.00 (0.72–1.41) for all subjects; None of the subgroup analyses were significant (smoking, HRT use, BMI, parity, vegetable consumption, multivitamin use, zinc, iron, calcium, ER status)	Age, total energy intake, education, race, HRT use, smoking, vegetable consumption, BMI, physical activity, alcohol consumption, age at first childbirth, multivitamin use, and mammography
Julin et al. (2012a) Sweden	Prostate cancer; the cohort of Swedish Men;41,089 men,45-79yr; 10.8yr F/U; 3,085 cases	FFQ ; 96-item FFQ; The mean estimated energy-adjusted cadmium intake = 19 µg/day (33% bread, 18% potatoes 15% other cereals than bread); T1 (<17), T2 (17–20), T3 (>20)	[T3 vs. T1] RR = 1.13 (1.03–1.24) for total; RR = 1.29 (1.08–1.53) for all localized prostate cancer; RR = 1.55 (1.16–2.08) or RR = 1.45 (1.15–1.83) for localized prostate cancer with a small waist circumference (<94 cm) or smoking, respectively	Age, family history of prostate cancer, education, BMI, waist circumference, physical activity, smoking, total energy intake, alcohol consumption, selenium, lycopene and calcium.
Julin et al. (2012b) Sweden	Breast cancer; 55,987 postmenopausal women; 12.2yr F/U; 2,112 cases (1626 ER+ and 290 ER-); Swedish Mammography cohort	FFQ; 67-item FFQ; the mean estimated energy-adjusted cadmium intake =15 µg/day (whole grain 31%, refined grain 20%, potatoes 18%, vegetables, 12%); T1 (<13), T2 (<13−16), T3 (>16)	[T3 vs.T1] •Among all postmenopausal women: RR = 1.21 (1.07–1.36) for all invasive tumors; RR = 1.19 (1.03–1.36) for ER+ tumor; RR = 1.33 (0.95–1.87) for ER- tumors;•Among lean and normal weight (BMI, 18.5-25 kg/m^2^): RR = 1.25 (1.05–1.49) for all invasive tumors; RR = 1.25 (1.03–1.52) for ER+ tumors; RR = 1.22 (0.76–1.93) for ER-tumors	Age, height, BMI, education, use of oral contraceptives, use of postmenopausal hormones, age at menarche, age at menopause, parity, age at first birth, alcohol consumption, glycemic load, total energy intake, and intake of whole grain and vegetables.
Sawada et al. (2012) Japan	All kinds of cancer; the Japan Public Health Center-based Prospective Study, 2 cohorts, cohort I, cohort II; 90,383 Japanese men and women, 45-74yr; 5 yr F/U, 5,849 cancer cases; 3,586 in men	FFQ; 138-item; rice 56%, wheat 11%, soybeans 13%, vegetables 20% ; the average estimated energy-adjusted cadmium intake 26.5 µg/day; men Q1 (18.4), Q2 (24.3), Q3 (29.3), Q4 (37.5); women Q1 (18.1), Q2 (22.9), Q3 (27.1), Q4 (33.9)	[Q4 vs. Q1] HR=0.94 (0.82–1.08) for men; HR=0.96 (0.81–1.15) for women; no site-specific cancers were associated with cadmium intake in men or women	Age, area, BMI, smoking, frequency of alcohol intake, leisure-time physical activity, and intake of meat, soybean, vegetable, and fruit. Further adjusted for menopausal status and use of exogenous female hormones in women.

ER, estrogen receptor; FFQ, food-frequency questionnaire; F/U, follow-up; Q, quartile; T, tertile; OR, odds ratio; RR, rate ratio; HR, hazard ratio.

**Figure 2:**
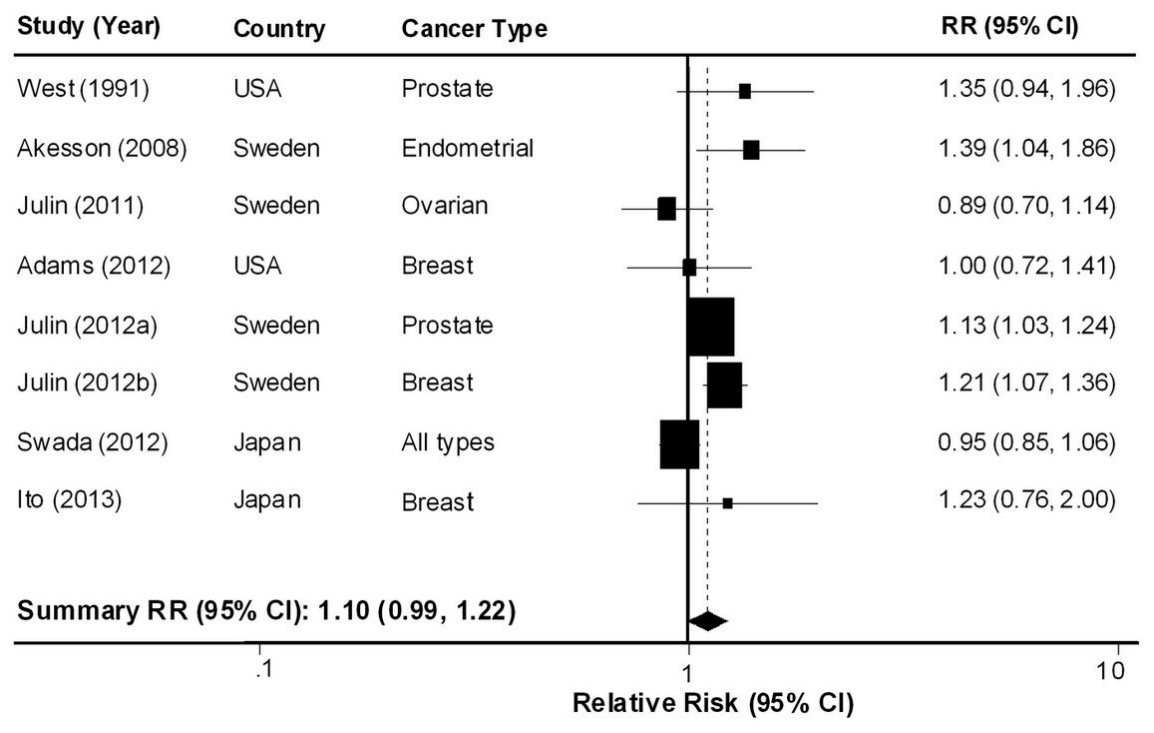
Forest plot for the association between dietary cadmium intake and cancer risk using a random effects model.

Figure **2** summarizes the associations between dietary cadmium intake and the risk of developing cancer in the eight studies. In the meta-analysis including all eight studies, we observed a significant heterogeneity (*P* = 0.020; I^2^ = 58%). Therefore, we calculated the summary estimate using a random effects model. Overall, dietary cadmium intake showed no statistically significant association with cancer risk (RR = 1.10; 95% CI: 0.99–1.22, for highest vs. lowest dietary cadmium group). To identify the source of heterogeneity, we conducted subgroup analyses based on study design, geographic location, and cancer type (Table **3**). In subgroup analyses, we summarized the data using a fixed effects model if the heterogeneity for each cancer type was not significant. In subgroup analyses by geographic location, we observed a positive association between dietary cadmium intake and cancer risk among studies conducted in Western countries (RR = 1.15; 95% CI: 1.08–1.23). We found an increased risk of prostate (RR = 1.14; 95% CI: 1.04–1.24), breast (RR = 1.15; 95% CI: 1.04–1.28), and endometrial cancers (RR = 1.40; 95% CI: 1.06–1.84) in the highest dietary cadmium group compared with the lowest dietary cadmium group.

**Table 3:** Subgroup analysis of the association between cadmium intake and cancer risk.

**Subgroup**	**No. of Study**	***P* for heterogeneity**	**RR (95% CI**)**^a^**
All	8	0.043	1.10 (0.99–1.22)
**Study design**			
Case-control	2	0.764	1.31 (0.97–1.75)
Cohort	6	0.022	1.09 (0.97–1.22)
**Geographic location**			
Western	6	0.175	1.15 (1.08–1.23)
Asian	2	0.295	0.96 (0.84–1.10)
**Cancer type**			
Prostate cancer	3	0.623	1.14 (1.04–1.24)
Breast cancer	4	0.277	1.15 (1.04–1.28)
Endometrial cancer	2	0.881	1. %2 (1.06–1.84)

^a^ To calculate the summary RR (95% CI), we used the fixed or random effects model based on the results of Q-statistics.

**Figure 3:**
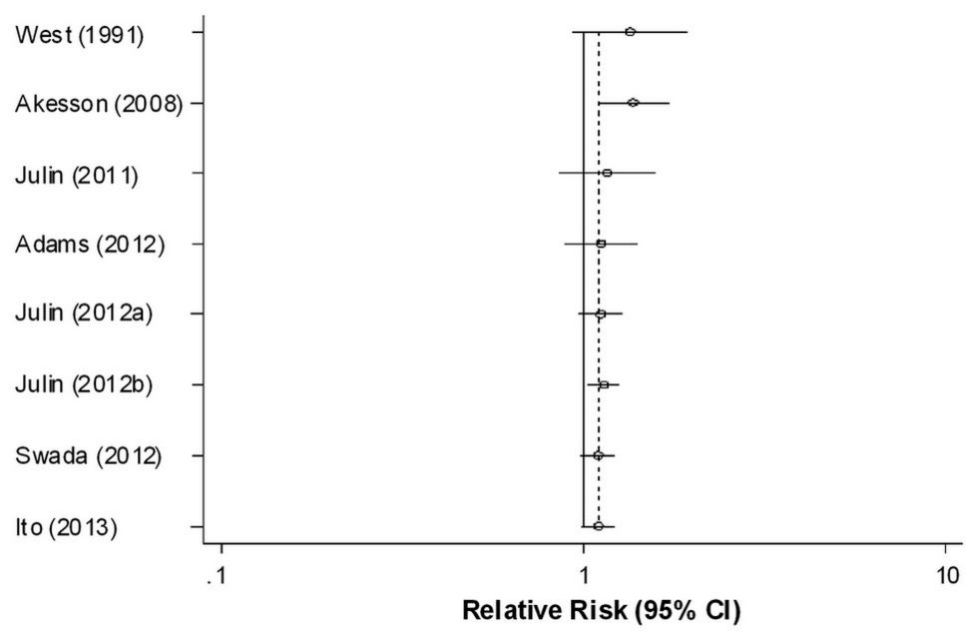
Cumulative meta-analysis.

**Figure 4:**
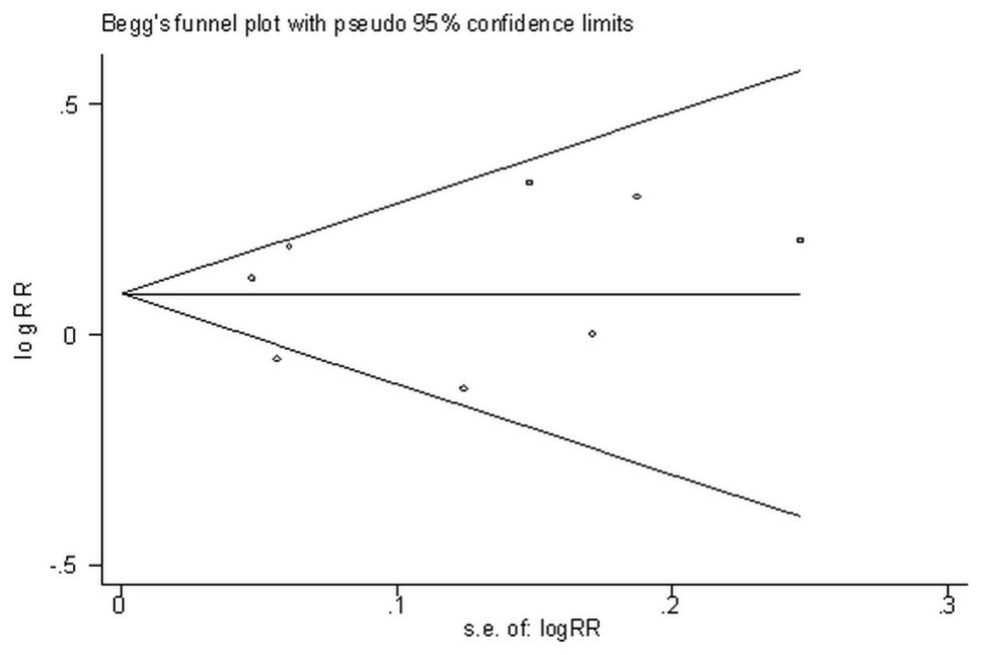
Publication bias.

We also conducted a cumulative meta-analysis based on publication year. The association between dietary cadmium intake and cancer risk did not change significantly as more data were collected (Figure **3**). Additionally, there was no influence of publication bias in our study (*P*=0.902 for the Begg test; *P*=0.713 for the Egger test). The funnel plot of studies was shown in Figure **4**.

## Discussion

Diet is the major source of cadmium for general populations that are not exposed to cadmium occupationally and do not live in cadmium polluted areas. We identified eight studies that investigated the role of dietary cadmium intake, but the findings of these studies are inconsistent. Several studies have investigated the role of cadmium in cancer development in female reproductive organs [9-12]. In a prospective cohort of 55,987 postmenopausal Swedish women, studies have reported significant positive associations between dietary cadmium and the risk of breast cancer [12]. However, no associations were found in a cohort study of 30,543 postmenopausal women in the United States [9] and a case-control study in Japan [16]. Other Swedish prospective studies found a positive association of cadmium and endometrial cancer [10] but found no association with ovarian cancer [11]. Some studies have investigated the role of dietary cadmium in prostate cancer [13,15]. A prospective cohort study of 41,089 Swedish men found an increased risk of prostate cancer among the highest cadmium exposure group [13]. However, another population-based case-control study in the United States reported no association [15]. In a Japanese cohort study of 90,383 individuals, Sawada et al. investigated the role of dietary cadmium in various types of cancer but found no associations [14]. In this meta-analysis, we found a positive association between dietary cadmium intake and cancer risk only among studies from Western countries. When compared internationally, the dietary cadmium intake of the general population was higher in Japan (26 µg/day) [20] than for the general populations in other countries (10-19 µg/day) [10,21]. These results suggest that the range of exposure affects the discrepancy of results [14,20]; when two Japanese studies were excluded, significant study heterogeneity disappeared. In addition, our results presented a possible association between dietary cadmium intake and hormone-related cancer risk. It is plausible that the exposure to cadmium and other metals over the past decades may partially explain the increased rates of these cancers in developed countries [6].

Many studies have investigated the role of cadmium in hormone-related cancers (e.g., breast and prostate cancer) in non-occupationally exposed populations using biomarkers [22-30] (Table **S1** and Table **S2**). Dietary cadmium intake was related to biomarkers for both long-term (e.g., urine) and recent (e.g., blood) exposures to cadmium [31]. Diet may be the major source of cadmium intake in these studies, thus the association between biomarkers of cadmium and cancer risk may indirectly indicate the role of dietary cadmium. Recent studies using biomarkers have supported consistently the role of low cadmium exposure in breast cancer risk [22-25]. In studies conducted in the United States, Gallagher et al. [22] and McElroy et al. [23] suggested that an increased risk for breast cancer is associated with elevated levels of urinary cadmium. Another case-control study assessing Lithuanian women reported that the mean cadmium levels in breast tumor tissue and urine were significantly higher in breast cancer patients [25]. More recently, a case-control study in Japan also found that a higher risk of breast cancer was associated with a higher cadmium level in urine (OR = 6.5; 95% CI: 2.91–12.62) [24]. Several studies have investigated the association between the detection of cadmium biomarkers and prostate cancer risk but report conflicting findings [26-30]. In an Italian hospital-based case-control study, Vincenti et al. [28] observed a strong positive association between the elevated risk of prostate cancer in subjects in the highest quartiles of toenail cadmium concentration compared with subjects in the bottom quartile (OR = 4.7; 95% CI: 1.3–17.5). However, no association was observed in a Taiwanese hospital-based case-control study using urinary cadmium [30] or in an American nested case-control study assessing levels of toenail cadmium [27].

It has been suggested that cadmium may induce cancer through multiple pathways, such as via aberrant gene expression, inhibition of oxidative stress, inhibition of DNA damage repair, or inhibition of apoptosis [7]. Recent studies have indicated that cadmium mimics the function of steroid hormones, such as estrogen and androgen, by binding and activating steroid receptors [6,32]. Based on the major role of these hormones in carcinogenesis in the reproductive system, this finding supports the potential role of cadmium in development of the hormone-related cancers [6], which is also observed in the present study. Studies using either *in vitro* cell culture or *in vivo* animal models provide evidence of the estrogenic or androgenic effects of cadmium on cell growth and gene expression [33-35]. Furthermore, several studies included in this meta-analysis indicate that the role of cadmium in the development of different cancers may be associated with its hormone mimicking properties [14]. Initially, dietary cadmium intake was only associated with the risk of an estrogen receptor positive (ER^+^) breast tumor but not an estrogen receptor negative (ER^-^) breast tumor in a cohort of postmenopausal women [12]. Several studies then found that stronger associations between cadmium and cancer risk were observed among individuals with low bioavailable estrogen such as low body mass index (BMI) [10,12,13] or nonusers of postmenopausal hormones [10]. However, cadmium may induce hormone-related cancers independently of its steroid-mimicking effects. Cadmium may facilitate other carcinogenic compounds to induce cancer at significantly lower levels than what would be normally required [7]. Additional experimental and epidemiological studies are required to verify the mechanism underlying the involvement of cadmium in the causal pathway of hormone-related cancers [6].

Several factors may influence the role of dietary cadmium in cancer development. Therefore, it is important to identify the high-risk population for cadmium-induced carcinogenesis―a population that is defined by increased exposure to cadmium or a higher absorbed dose of cadmium. First, dietary habits affect the level of cadmium exposure. Individuals who regularly eat crustaceans, mollusks, and cephalopods or consume large amounts of whole grains and vegetables have the highest exposure to dietary cadmium [3]. Although whole grain and vegetables are well known for their anti-carcinogenic effects [12], some studies have reported that these foods have conflicting roles in carcinogenesis [36,37]. The Health Professionals Follow-up Study reported that dietary whole grain intake was positively associated with prostate cancer risk [36]. The presence of cadmium in whole grains and vegetables may counteract their anti-carcinogenic effects and explain the absence of a protective association between vegetable consumption and the incidence of some hormone-related cancers. Second, the rate of intestinal absorption of cadmium may be affected by an individual’s nutritional status, as the rate of cadmium absorption is increased if a calcium, iron, or zinc deficiency is present [38]. The higher prevalence of iron depletion among women compared to men is likely a major cause for a higher body burden of cadmium among women [4,39]. Furthermore, smoking may increase the amount of cadmium exposure and thus affect the role of cadmium in carcinogenesis [10]. As a result, smokers, women with low iron levels, and people habitually consume foods rich in cadmium are at the highest risk of cadmium exposure and should be aware of their increased risk of having high cadmium levels.

To the best of our knowledge, this is the first meta-analysis combining the results of existing studies that have investigated the effect of dietary cadmium on cancer risk. Most are population-based cohort studies, which may eliminate recall and selection biases. However, we recognize several limitations in interpreting our results. The most important limitation is the validity of the estimated cadmium intake values. Estimates of dietary cadmium intake could vary widely among the populations studied based on the method of diet assessment and the cadmium database used [9]. FFQ may not accurately reflect food intake [40] and the actual absorbed dose of cadmium, as absorption of cadmium from ingested food may vary between individuals due to individual differences in nutritional status and the bioavailability of cadmium in various food items [38]. However, Julin et al. [41] validated the estimated dietary cadmium exposure in relation to biomarkers (i.e., cadmium in urine or blood). Most of the included studies are prospective; therefore, misclassification in our study is most likely non-differential, which may lead to the attenuation of the true association. Additionally, confounding factors such as co-exposure to other toxic chemicals and lifestyle factors (e.g., cigarette smoking) may affect the results. Finally, the small number of studies included in the meta-analysis limits the ability to draw a significant conclusion, especially in subgroup analyses. Most studies estimating the dietary cadmium exposure in relation to cancer risk have only been performed during the last few years, which make the number of studies available for this analysis limited.

## Conclusions

Our meta-analysis supports the findings of existing studies regarding the role of dietary cadmium intake in hormone-related cancer risk in Western countries. We cautiously suggest that chronic exposure to cadmium and other metalloestrogens may partly explain the risk of developing hormone-related cancers, particularly in Western populations. To reduce cadmium-induced cancer risk, it is important to identify the high-risk population (e.g., vegetarians; Fe-, Ca-, Zn-deficient individuals; smokers) and to provide an appropriate medical intervention. Although this meta-analysis of epidemiological studies suggests a link between cadmium and hormone-related cancers, more experimental and epidemiological studies using diverse populations are needed to establish a causal association, as well as to verify the underlying mechanisms.

## Supporting Information

Table S1 **The association between environmental cadmium exposure and breast cancer risk in studies using biomarkers.**
(DOCX)

Table S2 **The association between environmental cadmium exposure and prostate cancer risk in studies using biomarkers.**
(DOCX)
