# Supplementary material for: Dietary Cadmium Intake and the Risk of Cancer: A Meta-Analysis
Source: PLoS One. 2013 Sep 17;8(9):e75087. doi: 10.1371/journal.pone.0075087 (PMC3775812; doi:10.1371/journal.pone.0075087)
Supplement: Table S1 — The association between environmental cadmium exposure and breast cancer risk in studies using biomarkers. (DOCX) [file pone.0075087.s001.docx]

**Table S1. The association between environmental cadmium exposure and breast cancer risk using biomarkers.**

| **Study** | **Country** | **Study description** | **Biomarker** | **Outcome** |
| --- | --- | --- | --- | --- |
| Gallagher et al. (2010) | USA | Case-control study, women living on Long Island (2008-2009), 100 cases, controls; cross-sectional study, NHANES (1999-2008), 91 cases, 2,884 controls | Urine | Creatinine-adjusted UCd: Q1 (<0.22), Q2 (0.22−0.37), Q3 (0.37−0.60), Q4 (≥0.60); Li women: OR = 1.92 (0.77−4.77) for Q3 vs. Q1; OR = 2.69 (1.07−6.78) for Q4 vs.Q1; NHANES: OR = 2.50 (1.11−5.63) for Q3 vs. Q1; OR = 2.22 (0.89−5.52) for Q4 vs.Q1; *Data were adjusted for age, smoking, alcohol drinking, and menopausal status |
| McElroy et al. (2006) | USA | Population-based case-control study; 20-69yr women; 246 cases, 254 age-matched controls | Urine | Creatinine-adjusted UCd: Q4 (0.58 µg/g) vs. Q1 (< 0.26 µg/g); OR = 2.29 (1.3−4.2); *Data were adjusted for age, parity, age at first birth, family history of breast cancer, recent alcohol consumption, BMI, age at menarche, menopausal status, age at menopause, type of postmenopausal hormone use, education, and marital status. |
| Strymylaite et al. (2011) | Lithuania | Case-control study ; 57 cases, controls (benign tumor patients) | Breast tissue | The mean Cd concentration in breast cancer patients was 0.053 µg/g (95% CI: 0.042–0.065) for tumor sample and 0.02 µg/g (95% CI: 0.014–0.026) for healthy breast tissue sample (*P*<0.001). |
|  |  |  | Urine | Creatinine-adjusted UCd was significantly higher in cancer patients than in controls (*P* < 0.001). |
|  |  |  | Blood | No significant difference was found between the mean BCd level in cancer patients and controls (P>0.05). |
| Nagata et al. (2013) | Japan | Case-control study; 153 breast cancer cases, 431 controls | Urine | Creatinine-adjusted UCd: T2 (>2.62 µg/g) vs. T1 (<1.67 µg/g); OR = 6.05 (2.90−12.62); *Data were adjusted for age, education, age at menarche, number of births, age a first birth, BMI, smoking, alcohol intake, family history of breast cancer |

BCd, blood cadmium; OR, odds ratio; Q, quartiles; T. tertiles; UCd, urinary cadmium
