# Supplementary material for: Dietary Cadmium Intake and the Risk of Cancer: A Meta-Analysis
Source: PLoS One. 2013 Sep 17;8(9):e75087. doi: 10.1371/journal.pone.0075087 (PMC3775812; doi:10.1371/journal.pone.0075087)
Supplement: Table S2 — The association between environmental cadmium exposure and prostate cancer risk in studies using biomarkers. (DOCX) [file pone.0075087.s002.docx]

**Table S2. The association between environmental cadmium exposure and prostate cancer risk using biomarkers.**

| **Study** | **Country** | **Study description** | **Biomarker** | **Outcome** |
| --- | --- | --- | --- | --- |
| Ogunlewe et al. (1989) | Nigeria | Healthy Nigerian men (n=55), those with benign prostatic hypertrophy (BPH) (n=60), prostatic cancer (n=12) | Blood | The mean serum cadmium concentrations were 15.2 µmol/l ± 0.6, 15.5 µmol/l ± 0.7, and 24.2 µmol/l ± 0.9 for normal, BPH, and cancer subjects, respectively. |
|  |  |  | Prostate tissue | The mean prostatic tissue cadmium concentration for normal BPH and malignant glands were 3.8 µmol/g ± 0.6, 14.6 µmol/g ± 0.37. |
| Platz et al. (2002) | USA | Cohort study; nested in the prospective CLUE II study; 115 cases, 227 age-matched controls | Toenail | Men who have high toenail cadmium concentrations in the range observed in this general population sample were not at an increased risk for prostate cancer; Q5 (median 310.8 ppb) vs. Q1 (median 10.8 ppb): 0.70 (0.36−1.37) |
| Vinceti et al. (2007) | Italy | Case-control study; 45 cases, 58 controls | Toenail | Q4 (≥0.0306 µg/g) vs. Q1 (<0.0073 µg/g); OR = 4.7 (1.3−17.5); *Data were adjusted for BMI, socio-economic status, smoking, family history of prostate cancer, dietary intake of nutrients and toenail concentration of selenium, copper and zinc |
| Van Wijngaarden et al. (2008) | USA | Cross-sectional; 320 men, NHANES population sample | Urine | Among men with zinc intake less than the median level of 12.67 mg/day, an increase in 1 µg/g creatinine cadmium exposure was associated with a 35% increase in PSA level. |
| Chen et al. (2009) | Taiwan | Case-control study; 261 cases, 267 controls | Blood | No difference in blood Cd levels between cases and controls (median, 0.88 vs 0.87 µg/l, *P* = 0.45), but cases with higher BCd levels tended to be at more advanced stages. |
|  |  |  | Urine | Cases has lower urinary Cd level than controls (median 0.94 vs. 1.40 µg/g creatinine, *P* = 0.001), but cases with higher UCd levels tended to be at more advanced stages. |

BCd, blood cadmium; OR, odds ratio; Q, quartiles; UCd, urinary cadmium
